# A robust HIV-1 viral load detection assay optimized for Indian sub type C specific strains and resource limiting setting

**DOI:** 10.1186/0717-6287-47-22

**Published:** 2014-05-30

**Authors:** Arpan Acharya, Salil Vaniawala, Parth Shah, Harsh Parekh, Rabindra Nath Misra, Minal Wani, Pratap N Mukhopadhyaya

**Affiliations:** Dr. D. Y. Patil Biotechnology & Bioinformatics Institute, Tathawade, Pune INDIA; SN Genelab, 2nd Floor, President Plaza, Tower A, Nanpura, Ring Road, Surat, Gujarat India; Supratech Micropath Laboratory & Research Institute, Kedar Building, Ellisbridge, Opposite Krupa Petrol Pump, Parimal Garden, Ahmedabad, Gujarat India; Interdisciplinary Science, Technology and Research Academy, AISC, Hidayatullah Road, Camp, Pune, 411 001 Maharashtra India; Department of Microbiology, Padmashree Dr D Y Patil Medical College & Research Centre, Sant Tukaram Nagar, Pimpri, Pune, 411 018 Maharashtra India

**Keywords:** HIV-1, Gag, SYBR Green, RT PCR

## Abstract

**Background:**

Human Immunodeficiency Virus Type 1 (HIV-1) viral load testing at regular intervals is an integral component of disease management in Acquired Immunodeficiency Syndrome (AIDS) patients. The need in countries like India is therefore an assay that is not only economical but efficient and highly specific for HIV-1 sub type C virus. This study reports a SYBR Green-based HIV-1 real time PCR assay for viral load testing and is designed for enhanced specificity towards HIV-1 sub type C viruses prevalent in India.

**Results:**

Linear regression of the observed and reference concentration of standards used in this study generated a correlation coefficient of 0.998 (p < 0.001). Lower limit of detection of the test protocol was 50 copies/ml of plasma. The assay demonstrated 100% specificity when tested with negative control sera. The Spearman coefficient of the reported assay with an US-FDA approved, Taqman probe-based commercial kit was found to be 0.997. No significant difference in viral load was detected when the SYBR Green based assay was used to test infected plasma stored at -20°C and room temperature for 7 days respectively (Wilcoxon signed rank test, p = 0.105). In a comparative study on 90 pretested HIV-1 positive samples with viral loads ranging from 5,000–25,000 HIV-1 RNA copies/ml and between two commercial assays it was found that the later failed to amplify in 13.33% and 10% samples respectively while in 7.77% and 4.44% samples the copy number values were reduced by >0.5 log value, a figure that is considered clinically significant by physicians.

**Conclusion:**

The HIV-1 viral load assay reported in this study was found to be robust, reliable, economical and effective in resource limited settings such as those existing in India. PCR probes specially designed from HIV-1 Subtype C-specific nucleotide sequences originating from India imparted specificity towards such isolates and demonstrated superior results when compared to two similar commercial assays widely used in India.

## Background

At present estimating HIV-1 RNA plasma concentration is the standard of care in monitoring the progression of HIV/AIDS and is believed to have clinically useful prognostic value [1, 2]. Three different commercial assays [NucliSens HIV-1 QT RNA Assay (bioMérieux, France), Quantiplex HIV-1 RNA 3.0 Assay (Bayer Diagnostics Division, USA) and COBAS TaqMan HIV-1 48 test (Roche Molecular Systems, USA)] presently dominate the HIV-1 viral load testing market but are prohibitively expensive [3].

Real time PCR technology is currently considered the gold standard for HIV-1 viral load detection because of its high assay specificity, sensitivity and wide linear range of detection [4]. TaqMan chemistry is widely popular and accepted method but in order to develop this on real time PCR platform for quantification of HIV-1 viral RNA, three conserved regions within the viral genome are required all in close proximity which is often difficult due to the heterogenic genomic structure of HIV-1. The problem of sub-optimal performance by TaqMan chemistry based commercial assay is mostly encountered in countries where non B subtype of HIV-1 is prevalent because these assays are widely validated in USA and Europe where HIV-1 subtype B dominates [3, 5]. In addition to the TaqMan chemistry, SYBR Green chemistry based real time PCR is also widely used for HIV-1 viral RNA quantification assays.

In this study, we report a real time RT-PCR assay specifically optimized for HIV-1 C subtype that uses SYBR Green chemistry for quantitative detection of HIV-1 viral RNA in human plasma. The assay is designed to target a conserved region within the gag gene of the viral genome and is less expensive compared to those which employ dual-labeled TaqMan probes. The protocol gave consistent results across a wide range of clinical samples.

## Results

### Linear range, detection limit and inter-assay precision

**Figure 1:**
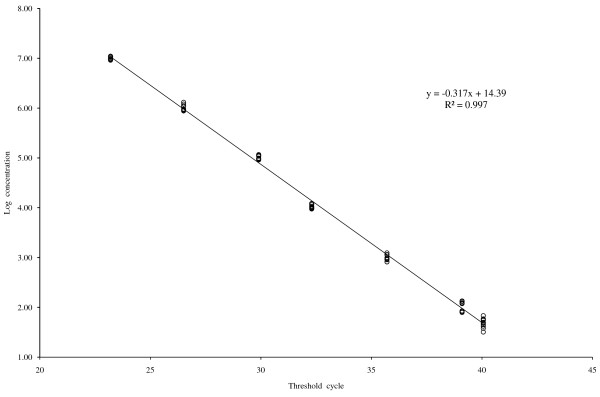
**Standard curve for quantification of HIV-1 viral load.** Log concentration of serial diluted reference standards plotted against their corresponding Threshold Cycles.

**Figure 2:**
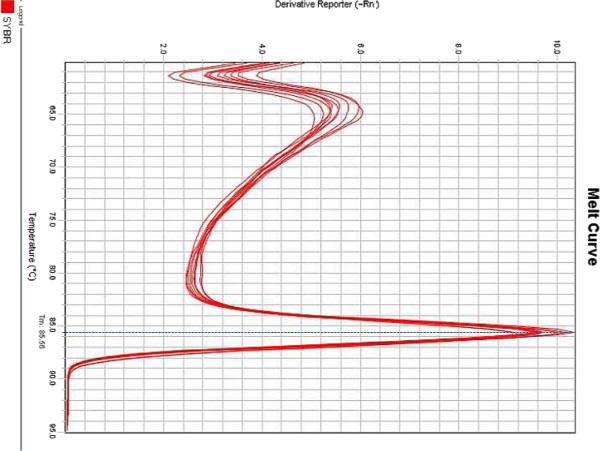
**Dissociation curve of the HIV-1 reference standards.** The negative first derivative of fluorescent signal (Y-axis) was plotted against Temperature (X-axis).

**Figure 3:**
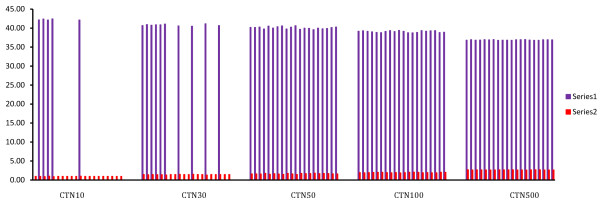
**Bar diagram used for finding the detection limit of the assay.** Log concentration of the dilution series plotted with their corresponding Threshold Cycles. Series 1: Threshold Cycles; Series 2: Log concentration of viral load; CTN10: 10 HIV-1 RNA copies/ml; CTN30: 30 HIV-1 RNA copies/ml; CTN50: 50 HIV-1 RNA copies/ml; CTN100: 100 HIV-1 RNA copies/ml; CTN500: 500 HIV-1 RNA copies/ml.

The linearity of the assay was determined using reference standards constructed specifically for this assay by using a recombinant plasmid harboring the amplified gag gene fragment as insert. Linear regression of the observed and reference concentrations yielded a correlation coefficient of 0.998 (p < 0.001). A standard curve was generated by plotting the threshold cycles of reference standards versus their log concentrations (Figure 1). The dissociation curve of all reference standards showed a melting temperature of 85.5°C (Figure 2).In order to compute the detection limit samples with known viral load in the range of 10 to 500 HIV-1 RNA copies/ml were analyzed. The results showed detectable amplification in 5 (25%) of the replicates for standard having 10 HIV-1 RNA copies/ml, in 10 (50%) of the replicates for those having 30 HIV-1 RNA copies/ml, and 20 (100%) having 50, 100 and 500 copies/ml of HIV-1 RNA respectively. The data is represented as a bar diagram where the log concentrations are compared with their corresponding threshold cycles (Figure 3). From this analysis, the lower limit of the assay was fixed at 50 copies/ml of HIV-1 RNA.

In order to evaluate the inter-assay precision of the test protocol the reference panel comprising of 25 samples with known HIV-1 viral load were tested in triplicates through 10 independent experiments. Statistical analysis of the data for standard deviation (SD) and coefficient of variation are shown in Table 1. The coefficient of variation was found to increase as the reference viral load approached detection limit of the assay.

**Table 1:** **Precision of the SYBR Green Real time PCR assay determined in this study**

Reference concentration (copies/ml)	Measured concentration (copies/ml)	Standard deviation (SD)	Coefficient of variation (CV)
52	50	1.65	0.0325
124	120	3.06	0.0251
344	345	0.47	0.0014
871	863	5.42	0.0063
1,235	1,250	10.14	0.0082
16,383	16,358	17.68	0.0011
267,816	267,098	507.70	0.0019
31,293	31,299	4.48	0.0001
541	534	4.71	0.0088
1,476	1,466	6.60	0.0045
4,572	4,555	12.26	0.0027
2571,210	2569,050	1,526.88	0.0006
76	89	9.43	0.1145
43,110	43,135	18.15	0.0004
5,447	5,414	23.10	0.0043
21,312	21,393	57.28	0.0027
96	94	1.65	0.0174
635,469	635,592	86.97	0.0001
626	620	4.48	0.0072
54,886	54,853	23.81	0.0004
8,742	8,781	27.58	0.0031
917,838	917,648	133.88	0.0001
74,511	74,552	29.23	0.0004
944	965	15.32	0.0161
9,648	9,984	237.82	0.0242

The standard deviation and the coefficient of variation were determined using data from 10 independent experiments.

### Sensitivity and specificity of the in-house HIV-I RT PCR assay

**Figure 4:**
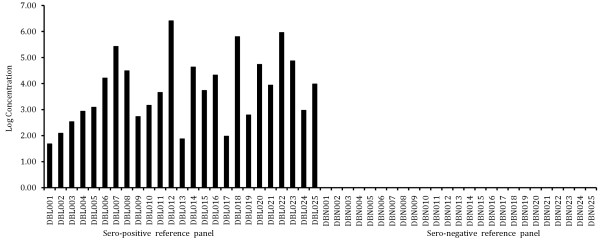
**Bar diagram showing sensitivity and specificity of the assay.** Sero-positive reference panel represented by DBL001 - DBLO25. Amplification of all samples in this panel indicates 100% sensitivity. Sero-negative reference panel represented by DBN001 – DBN025. No amplification in all samples in this panel indicates 100% specificity.

Clinical sensitivity of the assay was determined using samples from the reference panel. All 25 samples were found to be positive using the in-house SYBR Green RT PCR assay indicating 100% sensitivity. The specificity of the assay was also found to be 100% since none of the samples showed any detectable amplification from our reference panel of HIV-1-negative samples (Figure 4). This was further confirmed by analyzing the melting temperature profile of the amplicons using dissociation curve analysis. No significant fluorescent signals were detected from any non-specific amplification including primer dimers.

### Comparison of viral load measurements using the in-house and the US-FDA approved artus HIV-1 RG RT-PCR kit

**Figure 5:**
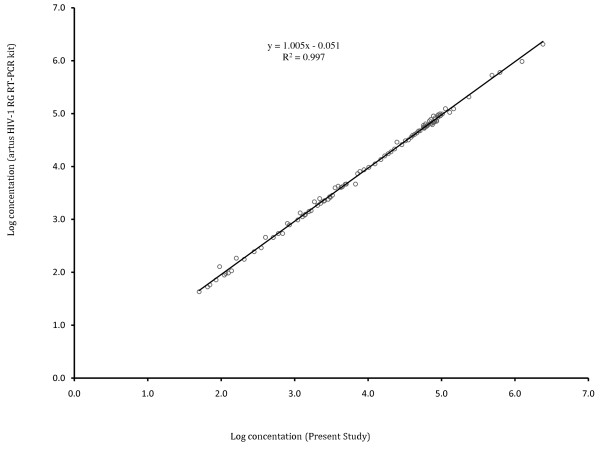
**Correlation of viral loads as determined by artus HIV-1 RG RT-PCR kit and Present Study.**

Viral load of 100 HIV-1 sero-positive samples was determined both by a commercially available US FDA-approved *artus* HIV-1 RG RT-PCR kit (Qiagen, Germany) as well as the SYBR Green RT PCR assay described in this study. The log concentration of HIV-1 RNA copies/ml of samples were compared by plotting results obtained using the present study protocol with those obtained using *artus* HIV-1 RG RT-PCR kit (Figure 5). The spearman coefficient of the in house assay with that of *artus* HIV-1 RG RT-PCR kit was found to be 0.997.

### Comparison of sensitivity with two commercial HIV-1 RNA detection assays

The detection limit of HIV RNA Real-Quant (Sacace Biotechnologies S.r.l, Italy) and HIV-1 Real Time RT PCR kit (Life River, Shanghai ZJ Biotech, China) are 500 and 1,000 HIV-1 RNA copies/ml respectively. Out of the 90 samples whose viral load varied between 5000 - 25,000 HIV-1 RNA copies/ml as determined by *artus* HIV-1 RG RT-PCR kit (Qiagen, Germany), 12 (13.33%) and 9 (10.0%) samples fail to show any detectable amplification when tested using HIV RNA Real-TM Quant (Sacace Biotechnologies S.r.l, Italy) and HIV-1 Real Time RT PCR kit (Life River, Shanghai ZJ Biotech, China) respectively. While 7 (7.77%) and 4 (4.44%) samples showed underestimation of viral load at a value that is greater than 0.5 log when compared with reference panel results. All the above mentioned samples had comparable results when tested using the protocol described in this study as shown in Table 1.

### Effect of ambient temperature on stability of HIV-1 RNA

**Figure 6:**
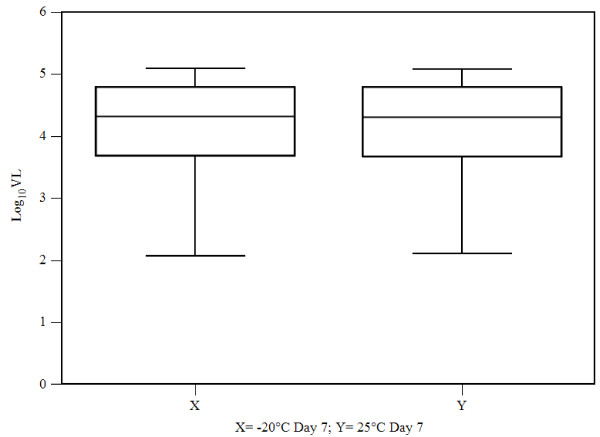
**Comparison of viral load from 10 plasma samples after storage at (X) -20°C degree and (Y) room temperature (25°C) for 7 days prior to SYBR Green Real Time PCR assay presented in a box and whisker plot.**

Effect of storage of plasma at room temperature was studied by analyzing 10 plasma samples each stored in (1) -20°C freezer and (2) room temperature (25°C) for 7 days. These samples were subjected to viral load testing and each reaction was performed in triplicate. Results indicate a w- value of 11.5, mean difference of 1.01, sum of positive ranks equaling to 43.5 and sum of negative ranks equaling to 11.5 respectively. As the critical value of w for n = 10 (p ≤ 0.05) was 8, the result was not significant meaning that there was no significant difference in viral load detected in both the groups (Wilcoxon signed rank test, p = 0.105) (Figure 6). The box and whisker plot in Figure 6 show differences between medians (DBM), over all visible spread (OVS) and DBM as a percentage of OVS within the two groups equaling to 0.013, 1.114 and 1.17% respectively indicating no significant difference between the two data sets.

## Discussion

At present SYBR Green chemistry based real time PCR is widely used for development of sensitive and economical quantitative PCR assays due to its competitive performance when compared to TaqMan chemistry based counterpart tests. In this study we described a SYBR Green chemistry based sensitive real time PCR assay for quantitative detection of HIV-1 viral load in human plasma samples.

Two assay panels were created for the study. The reference study panel comprised of 25 HIV-1 sero-positive samples with reference viral load assigned to them using *artus* HIV-1 RG RT-PCR kit (Qiagen, Germany) and an equal number of samples from healthy individuals. This panel was primarily used for development of the assay. The clinical study panel on the other hand was a secondary one comprising of 100 samples and was used to evaluate the performance of the assay. Reference viral load values were also assigned to this panel using *artus* HIV-1 RG RT-PCR kit (Qiagen, Germany).

Most commercial HIV-1 viral load assays are designed and optimized for the detection of viruses found in North America and Europe. As a result, these assays either under-quantify or fail to amplify some of the HIV-1 non-B subtypes [6–8]. This observation motivated us to identify the gag gene from Indian subtype C HIV-1 viruses as the target to develop this SYBR Green chemistry based HIV-1 viral load estimation assay. The linearity of our assay ranged from 50 HIV-1 RNA copies to 10^7^ HIV-1 RNA copies/ml of plasma sample and had a lower limit of detection of 50 HIV-1 RNA copies/ml.

The normalized measure of dispersion of the probability distribution of data generated from inter assay precision testing and expressed as coefficient of variation was found to increase as the values came closer to the detection limit. This was found to be acceptable since the linear range of detection was wide and at par with standard quantitative real time PCR assays [9–11].

The sensitivity of the assay was found to be 100%. No amplification in 25 clinical samples from HIV-1 sero negative reference panel indicated 100% specificity. This finding was further consolidated by dissociation curve analysis of all the sero positive reference panel samples. Sharp peaks at 85.5°C were detected in all sero positive reference panel samples and was absent in samples from healthy individuals. The results obtained from clinical study panel indicated a high level of concordance between the viral loads generated using the commercial kit and the assay described in this study.

The poor sensitivity of two commercial assays [HIV RNA Real-TM Quant (Sacace Biotechnologies S.r.l, Italy) and HIV-1 Real Time RT PCR kit (Life River, Shanghai ZJ Biotech, China) respectively] shown in this study may be due to presence of nucleotide mismatches within the primer and probe binding regions of HIV-1 genome. We believe that suboptimal attention towards HIV-1 subtype C specific sequences originating from India was the primary reason for such results. This finding is in line with data presented by Drexler et al. which demonstrated that HIV-1 positive samples which were reported negative by three commercial assays were shown to have a HIV-1 viral load of >5,000 copies/ml when tested using a home brew real time PCR protocol [7].

To evaluate the performance of the assay in a resource limited setting we used the Wilcoxon signed-rank test (similar to the paired student’s *t*-test) as a non-parametric statistical hypothesis test to compare the two related samples (plasma samples stored in -20°C and at room temperature respectively) and ascertain whether their population mean ranks differed. Results indicated that there were no significant differences in viral loads detected in both the groups (Wilcoxon signed rank test, p = 0.105) thus confirming the conclusions drawn from similar studies undertaken elsewhere [12]. In resource limiting setting like India cold chain facility is not always available for transportation of plasma samples from collection centers to the reference laboratories. Stability of the samples at ambient temperature is therefore important for proper clinical management of the patients.

## Conclusions

There has been a significant increase in sensitivity of HIV-1 RNA quantification due to systematic involvement of advanced molecular techniques over the years. However, enhanced cost and demand for greater reagent and instrument sophistication is a necessary evil that needs to be traded off to achieve high level of accuracy and consistency. This is therefore a compelling reason for exploring areas where economy and ease of work can be linked to accuracy, sensitivity and specificity in HIV-1 RNA quantification. To address these issues we developed and evaluated the performance of SYBR Green chemistry based HIV-1 viral load detection assay. The significance of this study is high for a developing country like India which has a high rate of HIV-1 infection. Some commercial assays originating from Europe and China with poor attention to India-specific HIV-1 Subtype C genome sequences generated sub optimal data in our study and therefore carry a risk of generating error prone data with potential to mislead therapeutic decisions. In these contexts the assay described in this study specifically designed for Indian Subtype C viruses is useful, economical and reliable for routine monitoring of HIV-1 viral load in resource limited settings.

## Methods

### Clinical sample collection and processing

The reference panel comprised of blood samples collected from 25 each of HIV-1 sero-positive (drug naïve) and sero-negative (with no AIDS related risk factors) individuals from India in K_2_-EDTA vacutainer tubes (Becton Dickinson, San Diego, California, USA) after obtaining written consent from the donors. The status of HIV-1 infection was confirmed by (a) Tridot rapid assay (J. Mitra & Co. Ltd., New Delhi, India) (b) multiple enzyme-linked immunosorbent assays (Biochem Immunosystems, Montreal, Canada) and (c) western blot analysis (LAV Blot HIV-1, Bio- Rad, France). The viral load for each of these samples was determined using a US FDA-approved kit (*artus* HIV-1 RG RT-PCR kit; Qiagen, Germany) and used as reference concentration.

Plasma were separated from the blood samples within 4 hours of collection and stored in a -20°C freezer. Details of the study participants are described in Table 2.

**Table 2:** **Characteristics of reference study panel**

Sero positive reference panel (n = 25)			Sero negative reference panel (n = 25)		
Sample ID	Age	Gender	Sample ID	Age	Gender
DBL001	28	Male	DBN001	29	Male
DBL002	39	Female	DBN002	24	Female
DBL003	44	Male	DBN003	21	Male
DBL004	21	Male	DBN004	46	Male
DBL005	47	Male	DBN005	35	Male
DBL006	24	Female	DBN006	39	Female
DBL007	27	Female	DBN007	42	Female
DBL008	35	Male	DBN008	31	Male
DBL009	45	Male	DBN009	36	Male
DBL010	54	Male	DBN010	45	Female
DBL011	19	Female	DBN011	26	Male
DBL012	28	Male	DBN012	21	Female
DBL013	34	Female	DBN013	37	Male
DBL014	31	Male	DBN014	41	Male
DBL015	25	Female	DBN015	28	Male
DBL016	42	Male	DBN016	37	Female
DBL017	47	Male	DBN017	35	Male
DBL018	29	Female	DBN018	32	Female
DBL019	24	Male	DBN019	27	Male
DBL020	27	Female	DBN020	26	Female
DBL021	29	Female	DBN021	29	Female
DBL022	36	Female	DBN022	38	Male
DBL023	34	Female	DBN023	39	Female
DBL024	31	Male	DBN024	34	Male
DBL025	50	Male	DBN025	38	Male

The clinical study panel comprised of blood samples collected from 100 HIV-1 sero-positive individuals. The viral load for each of these samples was determined using a US FDA-approved kit (*artus* HIV-1 RG RT-PCR kit; Qiagen, Germany) and used for analysis and comparison.

The study program was duly approved by a bio-safety and bio-ethics committee (Approval number SNGL/2012/GY06) overseeing ethical aspect of such research programs within the organization.

### Oligonucleotide primer design

In order to avoid primer mismatch the most conserved region of HIV-1 gag gene was chosen for designing of the oligonucleotide primers. This region was identified by analyzing several full length HIV-1 gag gene nucleotide sequences submitted from India and obtained from NCBI GenBank database (http://www.ncbi.nlm.nih.gov/genbank) [13]. The sequences were aligned with the help of multiple sequence alignment program ClustalW v.2 [14] and conserved regions identified were used to manually design PCR primers. Five different sets of forward and reverse primers were designed, their reaction kinetics as well as self-complimentarity checked using Primer Express 3.0 program (Life Technologies, USA) and experimentally evaluated for their efficiency in amplifying target DNA.

### Construction of reference standards

For construction of reference standards, the HIV-1 gag gene PCR fragment generated using the newly designed PCR primers was cloned in the pCR4-TOPO vector (Life Technologies. USA) following manufacturer’s instructions. The recombinant plasmid was purified and identity of the cloned insert confirmed by double strand DNA sequencing using the BigDye Terminator v3.1 Cycle Sequencing Kit and ABI PRISM® 3500 Dx Genetic Analyzer (Applied Biosystems, USA).

In order to generate quantization standard, the recombinant plasmid was serial diluted and calibrated against reference standards of the US FDA approved *artus* HIV-1 RG RT-PCR kit (Qiagen, Germany). A seven dilution series of the reference standard was prepared with copy numbers as follows: 10^1^, 10^2^, 10^3^, 10^4^, 10^5^, 10^6^ and 10^7^ HIV-1 viral RNA copies/ml.

### RNA extraction and reverse transcription–real time PCR

HIV-1 RNA was extracted from plasma samples using a QIAamp Viral RNA mini kit according to manufacturer’s instructions (Qiagen, Germany). SYBR Green I - based one-step real time quantitative RT-PCR amplification was performed using a StepOne Plus Real Time PCR System (Applied Biosystems, USA). Fifty μL of a reaction mixture comprised of 25 μL of 2X QuantiFast SYBR Green RT-PCR master mix (Qiagen, Germany) which contained HotStarTaq DNA Polymerase, 0.5 μL 100X QuantiFast RT Mix, 20 μL of RNA and each primers (forward and reverse) at a final concentration of 5 pmoles per reaction.

The nucleotide sequence of the primers used in this study is as follows: 5′- ACATCAAGCAGCCATGCAAAT - 3′ (forward) and 5′- TACTAGTAGTTCCTGCTATGTC - 3′ (reverse). ROX dye was used as a passive reference to normalize fluorescent signals. The thermal cycling profile comprised of 30 minutes of reverse transcription at 45°C, 5 minutes of heating at 95°C to activate HotStart *Taq* DNA Polymerase followed by 45 cycles of PCR amplification each comprising of 95°C for 15 seconds, 55°C for 15 seconds, and 72°C for 30 seconds. Data collection was done at 72°C of each cycle. Following amplification, a melting curve (dissociation curve) analysis was performed to verify the authenticity of the amplified products by checking their specific melting temperatures (Tm). In each batch all reference standards as well as samples were tested in triplicate and appropriate negative as well as reagent controls were included to exclude the possibility of cross contamination during the setting up of reactions.

### Intra- and inter-assay validation

For intra- and inter-assay validation 25 HIV-1 sero-positive samples were tested 10 times each in different batches independently and in triplicate including the reference standards and controls and their coefficient of variation was calculated.

### Sensitivity and specificity

For finding limit of detection of the assay 10, 30, 50, 100 and 500 copies/ml of serially diluted standards were tested in triplicate in 20 independent experiments. In order to measure the specificity of the assay all known HIV-1 sero-negative samples (Table 2) were subjected to real time PCR amplification followed by comparison of the melting-temperature data with known positives as well as no-template control (NTC) samples.

### Comparing sensitivity of detection against two commercial HIV-1 copy number detection assays

A panel of 90 plasma samples which was a part of the study panel and with HIV-1 viral copy number ranging from 5,000–25,000 HIV-1 RNA copies/mL as estimated using *artus* HIV-1 RG RT-PCR kit (Qiagen, Germany) and confirmed as sub type C using method described by Siddappa et al. [15] were simultaneously subjected to quantitative detection of HIV-1 RNA using (a) HIV RNA Real-TM Quant (Sacace Biotechnologies S.r.l, Italy) (b) HIV-1 Real Time RT PCR kit (Life River, Shanghai ZJ Biotech, China) and (c) assay method developed in this study. All RT-PCR reactions were set in duplicate in two separate thermal cyclers of identical make (StepOne Plus Real Time PCR System; Applied Biosystems, USA) in order to minimize the influence of experimental errors.

### Effect of storage time and temperature on stability of plasma samples

Plasma samples of 10 patients with known viral load were collected and two aliquots were made from each of them. One set of plasma were stored at -20°C and the other at 25°C. RNA was extracted from both set of samples at day seven and their viral load estimated in triplicate using the method described in this study.

### Statistical analysis

The linearity of the assay was determined by linear regression method using the MedCalc v.12.7.2 (Acacialaan 22, B-8400 Ostend, Belgium) program. Sensitivity of the assay was expressed as the percentage of samples that scored positive using the protocol described in this study divided by the total number of samples that scored positive using an US -FDA approved commercial assay. Specificity of the assay was calculated as the number of negative samples scored using the present study divided by the total number of known negative samples obtained from healthy donors and was expressed in percentage. Performance of the assay was measured by doing a comparative analysis with *artus* HIV-1 RG RT-PCR kit (Qiagen, Germany) and applying the Spearman correlation coefficient formula (http://www.wessa.net/rankcorr.wasp). The effect of ambient temperature on the stability of HIV-1 RNA was analyzed using the Wilcoxon signed rank test [16] and the box and a whisker plot was created using BioStat program (AnalystSoft Inc, USA).
